# Assessing PD-L1 expression in non-small cell lung cancer and predicting responses to immune checkpoint inhibitors using deep learning on computed tomography images

**DOI:** 10.7150/thno.48027

**Published:** 2021-01-01

**Authors:** Panwen Tian, Bingxi He, Wei Mu, Kunqin Liu, Li Liu, Hao Zeng, Yujie Liu, Lili Jiang, Ping Zhou, Zhipei Huang, Di Dong, Weimin Li

**Affiliations:** 1 Department of Respiratory and Critical Care Medicine, Lung Cancer Treatment Centre, West China Hospital, West China Hospital, Sichuan University, Sichuan, China.; 2 School of Electronic, Electrical and Communication Engineering, University of Chinese Academy of Sciences, Beijing, China.; 3 CAS Key Laboratory of Molecular Imaging, Beijing Key Laboratory of Molecular Imaging, The State Key Laboratory of Management and Control for Complex Systems, Institute of Automation, Chinese Academy of Sciences, Beijing, China.; 4 Beijing Advanced Innovation Center for Big Data-Based Precision Medicine, School of Medicine, Beihang University, Beijing, China.; 5 Department of clinical medicine, Sichuan vocational college of health and rehabilitation, Zigong, Sichuan, China.; 6 Department of Respiratory Medicine, West China-Guang'an Hospital, Sichuan University, Sichuan, China.; 7 Department of Respiratory and Critical Care Medicine, West China Hospital, Sichuan University, Chengdu, Sichuan, China.; 8 Department of Pathology, West China Hospital, Sichuan University, Chengdu, Sichuan, China.; 9 School of Artificial Intelligence, University of Chinese Academy of Sciences, Beijing, China.; 10 Zhuhai Precision Medical Center, Zhuhai People's Hospital (affiliated with Jinan University), Zhuhai, China.

**Keywords:** PD-L1 expression, deep learning, computed tomography, immunotherapy, non-small cell lung cancer

## Abstract

**Rationale:** This study aimed to use computed tomography (CT) images to assess PD-L1 expression in non-small cell lung cancer (NSCLC) and predict response to immunotherapy.

**Methods:** We retrospectively analyzed a PD-L1 expression dataset that consisted of 939 consecutive stage IIIB-IV NSCLC patients with pretreatment CT images. A deep convolutional neural network was trained and optimized with CT images from the training cohort (n = 750) and validation cohort (n = 93) to obtain a PD-L1 expression signature (PD-L1ES), which was evaluated using the test cohort (n = 96). Finally, a separate immunotherapy cohort (n = 94) was used to assess the prognostic value of PD-L1ES with respect to clinical outcome.

**Results:** PD-L1ES was able to predict high PD-L1 expression (PD-L1 ≥ 50%) with areas under the receiver operating characteristic curve (AUC) of 0.78 (95% confidence interval (CI): 0.75~0.80), 0.71 (95% CI: 0.59~0.81), and 0.76 (95% CI: 0.66~0.85) in the training, validation, and test cohorts, respectively. In patients treated with anti-PD-1 antibody, low PD-L1ES was associated with improved progression-free survival (PFS) (median PFS 363 days in low score group vs 183 days in high score group; hazard ratio [HR]: 2.57, 95% CI: 1.22~5.44; *P* = 0.010). Additionally, when PD-L1ES was combined with a clinical model that was trained using age, sex, smoking history and family history of malignancy, the response to immunotherapy could be better predicted compared to either PD-L1ES or the clinical model alone.

**Conclusions:** The deep learning model provides a noninvasive method to predict high PD-L1 expression of NSCLC and to infer clinical outcomes in response to immunotherapy. Additionally, this deep learning model combined with clinical models demonstrated improved stratification capabilities.

## Introduction

Lung cancer remains the leading cause of cancer mortality worldwide 1. Non-small cell lung cancer (NSCLC) represents 80-85% of primary lung malignancies 2. The treatment strategy for NSCLC has rapidly evolved with the introduction of immune checkpoints inhibitors (ICIs) targeting PD-1 or PD-L1, but durable disease response remains limited to a subset of patients. Given the overall response rates to ICI treatment, ranging from 14% to 20% in unselected patients 3,4, it is essential to identify predictive biomarkers for the selection of patients who are more likely to respond to immunotherapy.

PD-L1, which is expressed by cells in the tumor microenvironment, engages PD-1 on T cells and triggers inhibitory signaling of the T cell receptor, reducing T-cell killing capacity and blocking effector functions 5. PD-L1 expression on tumor cells is induced by constitutive oncogenic signaling 6. Alternatively, it can be induced in response to immune-stimulating cytokines, such as interferons, that are produced by an active antitumor immune response 7,8. Currently, PD-L1 expression on tumor cells assessed by immunohistochemistry is the only approved diagnostic biomarker for immunotherapy in patients with NSCLC and has been convincingly demonstrated to be associated with the efficacy of ICIs in NSCLC 9,10. In the first-line setting, pembrolizumab monotherapy demonstrated improved progression-free survival (PFS) and overall survival (OS) benefit compared to chemotherapy in NSCLC patients with PD‐L1 expression on ≥ 50% of tumor cells (KEYNOTE 024) 9,11. According to a recent study, though PFS and OS were significantly longer in the pembrolizumab-treated group than in the chemotherapy-treated group in the full NSCLC cohort with PD-L1 ≥ 1%, a clear PFS and OS benefit was only observed in the subgroup of patients with PD-L1 ≥ 50%, and higher PD-L1 expression corresponded to greater benefit (KEYNOTE 042) 12. The FDA approved immunohistochemistry assay for PD-L1 expression, utilizing a cut-off of 50% tumor proportion score (TPS) for first-line treatment with pembrolizumab. Pembrolizumab monotherapy is preferred for patients with stage IV NSCLC and PD-L1 levels of 50% or more who are negative for *EGFR* mutations and *ALK* fusions. However, expression levels of PD-L1 are intratumorally heterogeneous and dynamic by immunohistochemistry analysis with different antibodies and platforms, as well as multiple scoring criteria, complicating interpretation of the results 13-15. Therefore, a noninvasive and whole-tumor-based biomarker is urgently needed.

Radiological images are routinely available in clinical practice. Unlike traditional biopsy-based assays that represent only part of the tumor, images reflect information on the entire tumor burden in a non-invasive manner and avoid the effects of tumor heterogeneity 16,17. Radiomics is the science of quantifying patterns of tumor phenotypes on radiographic images in a high throughput manner and analyzing them with bioinformatics tools to build clinically relevant models that assess tumor and microenvironment heterogeneity 19. Indeed, radiomics-based biomarkers have shown success in predicting response to different treatments in different tumor types 21-26.

In this study, we aimed to develop a radiomic signature to predict PD-L1 TPS ≥ 50% and to evaluate its potential ability to predict clinical outcomes in anti-PD-1 immunotherapy-treated metastatic NSCLC patients.

## Methods

### Patients

This study was granted ethics approval by the institutional review board of the West China Hospital, Sichuan University (Approval number: 2019-612) and was performed in accordance with ethical standards of the 1964 Declaration of Helsinki and its later amendments. Medical records were retrieved to collect clinical data (age, gender, smoking status, and family history), treatment regime, and survival outcome. Informed consent was waived due to the retrospective nature of this study.

### PD-L1 expression dataset

A consecutive cohort of 2094 patients with stage IV NSCLC who underwent PD-L1 staining based on histological specimens from January 2016 to December 2018 at the West China Hospital of Sichuan University were retrospectively screened. Eight hundred twenty-four patients were excluded due to low-quality CT images. In this study, low-quality CT was defined as CT reconstructed with 30f reconstruction kernel. All included CTs were reconstructed with 60f kernel. Three hundred thirty-one patients were excluded due to insufficient clinical or pathological information. Finally, 939 patients were included in the analysis. Immunohistochemical (IHC) assays were performed using SP142 antibody on the Ventana Benchmark platform. Slides were scanned and independently scored by two pathologists who estimated the percentage of PD-L1 protein in both tumor cells and tumor infiltrating immune cells.

### Immunotherapy dataset

Patients who met the following inclusion criteria were included in this dataset: 1) histologically confirmed primary NSCLC; 2) stage IV; 3) receipt of pembrolizumab monotherapy (200 mg every 3 weeks at the approved dose) or pembrolizumab combined with chemotherapy as the first-line treatment; and 4) pretreatment CT and CT data during follow-up were available. Patients were excluded if they met one or more of the following criteria: 1) clinical data, including age, sex, smoking status and family history of tumor were missing; 2) other treatments, such as radiotherapy, surgery or herbs, were adopted during immunotherapy treatment; 3) low-quality CT images; or 4) lost to follow-up before disease progression. All enrolled patients submitted to CT scan two weeks before immunotherapy. The follow-up interval was 8-10 weeks, and routine laboratory tests and CT scans were performed during this time. Finally, 94 patients admitted between January 2017 to December 2018 from West China Hospital were included in the cohort. PFS was obtained using iRECIST criteria, which is the response criteria used in trials testing immunotherapeutic 27.

### Data preprocessing

This study consisted of two datasets, the PD-L1 expression dataset and the immunotherapy dataset. Both datasets contained 4 clinical characteristics (age, gender, smoking status, and family history), as well as CT images. The PD-L1 expression dataset was used to train the PD-L1 expression signature (PD-L1ES) program to reflect whether PD-L1 TPS is higher than 50%, while the immunotherapy dataset was used to evaluate the PFS utilizing the PD-L1ES program. In this experiment, we obtained the training, validation and test cohorts from the PD-L1 expression dataset by stratified and random sampling of patients at a ratio of 8:1:1. Details of the datasets and experimental flow chart are shown in **Supplementary Figure 1**.

To make full use of the enrolled data, the k-nearest neighbor (KNN) algorithm was first performed to impute missing clinical values. In detail, the Euclidean distance between patients was calculated using patients' nonmissing variables, and missing values were filled by the weighted average of the closest K patients' value (k = 3 in this study). Then, all clinical characteristics were normalized to make the model easier to learn. All CT images were resampled to the same resolution of 1×1×1 mm^3^.

### Delineation of the region of interest (ROI)

The region of interest (ROI) of the primary tumor lesion was delineated by an experienced respiratory medicine specialist on the workstation. Next, we performed rectangular clipping of the ROI area amplifying to 5 pixels around to avoid the bias of ROI area delineation. Additionally, the upper and lower slices of the ROI slice were also cut and used as training sample to amplify the amount of available data. Furthermore, extracting the upper and lower slices at the same time also mitigates the error introduced by the radiologists' selection of slices. Finally, to unify the input size, we resized all ROI patches into 112×112 pixels. To more fully describe the tumor, we extracted radiomic features of ROIs to improve the accuracy of the model.

### Establishment and evaluation of PD-L1 expression signature

**Figure 1:**
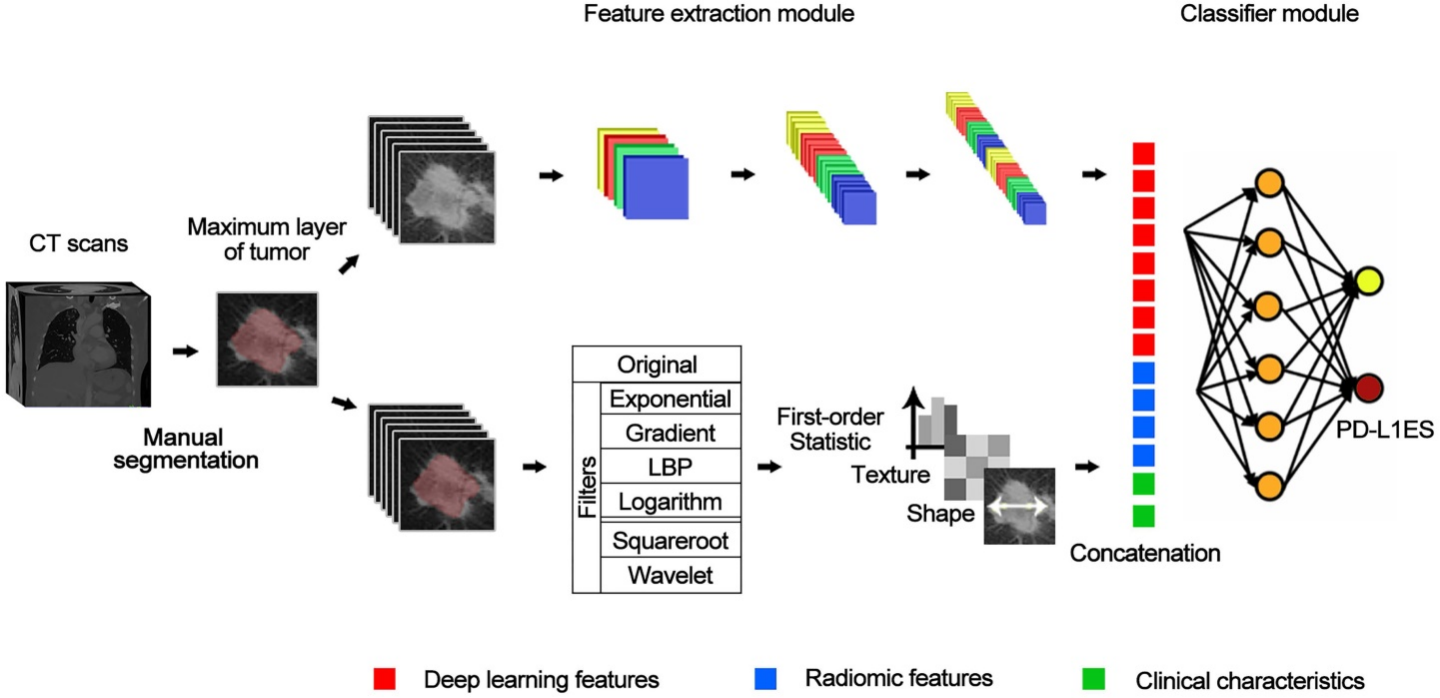
The structure of deep learning network. The entire network includes a feature extraction module (a convolutional network) for extracting deep learning features, and a classification module (a fully-connected network) for classifying PD-L1 expressions based on deep learning features combined with predefined radiomic features and clinical characteristics.

We built the PD-L1ES using a deep learning approach. The network (**Figure 1**) consisted of three parts, a deep learning feature extraction module based on the densenet121, a handcrafted conventional radiomic feature extraction module, and a classifier module based on the fully connected classification layer.

The input of the deep learning feature extraction module primarily included the slice of the CT tumor region. Before inputting the radiomic features, we employed the Mann Whitney U test to reduce the dimensionality of the radiomic features based on the training cohort. For clinical characteristics, we standardized them with z-score and concatenate them with radiomic features and deep learning features as the input of the fully connected layer. The output of the whole model represented the high expression probability and was regarded as PD-L1ES. During training of the model, cross-entropy loss function and Adam optimizer were used, and the learning rates were set to 1e-4, 1e-5 and 1e-6. We dropped the learning rate by 20 epochs per training. The best standard for training in each step was the best result of the validation cohort.

The predictive value of the PD-L1ES was evaluated with area under the receiver operating characteristic curve (AUC) and the decision curve. Meanwhile, we also compared PD-L1ES to a clinical model (CM; built using clinical characteristics only), a radiomic model (RM; built using only predefined radiomic features), and a deep learning (DL) model (only comprising deep learning features). In addition, we read the machine manufacturer, X-ray tube current and slice thickness from the CT metainformation and then analyzed the influence of these parameters on the PD-L1ES results. To verify the robustness of PD-L1ES, we randomly re-divided the validation and test cohorts and retrained twice. Furthermore, we used PD-L1ES obtained from the model to perform PFS prediction in the immunotherapy dataset. Meanwhile, we also used clinical features to establish a clinical model via Cox regression and performed comparative analysis with respect to PD-L1ES.

### Statistical analysis

PD-L1ES was built in the training cohort and verified in the testing cohort. During the training process, results of the adjustment were primarily determined by the results of the validation cohort. Delong test was used as a method to calculate the receiver operating characteristic curve (ROC) difference. Moreover, the predicted value of the binarized signature (using X-tile 28) in predicting the efficacy of immunotherapy was investigated with Kaplan-Meier curves and Log-rank test, which was further compared to the Kaplan-Meier curves obtained based on IHC of PD-L1 expression 28. We performed all analysis using R software (version 3.5.2; http://www.R-project.org) and Python (version 3.6.5, https://www.python.org/).

## Results

### Clinical characteristics

In the PD-L1 expression dataset, 939 patients were included. The mean age was 58.8 years (±10.7), and 576 (61.3%) patients were male. There were 432 (46.0%) never-smokers, and PD-L1 expression values were greater than 50% in 328 (34.9%) patients. Demographic and clinical characteristics of the PD-L1 expression dataset are shown in **Table 1.**

In the immunotherapy dataset, 77 patients (81.9%) were male, with a mean age of 60.7 years (± 11.1). Seventy-three (77.7%) were smokers, and the remaining 21 (22.3%) were nonsmokers. Fifty were diagnosed with adenocarcinoma, and the remaining included 33 patients with squamous cell carcinoma, 7 patients with neuroendocrine tumors, 2 patients with lymphoepithelioma-like carcinoma and 2 patients with undifferentiated NSCLC. Fifty-one (54.3%) received pembrolizumab monotherapy as the first-line treatment, and 43 (45.7%) received pembrolizumab combined with chemotherapy as the initial treatment. Clinical characteristics of the immunotherapy dataset are shown in **Supplementary Table 1.**

### Validation of PD-L1 expression signature in predicting PD-L1 expression

We extracted 1316 radiomic features that were calculated from images processed using different filters. Detailed information about the filters and features is shown in **Supplementary Table 2.** All radiomic features we extracted abided by the feature definitions that expand on the Imaging Biomarker Standardization Initiative (IBSI) 29.

**Figure 2:**
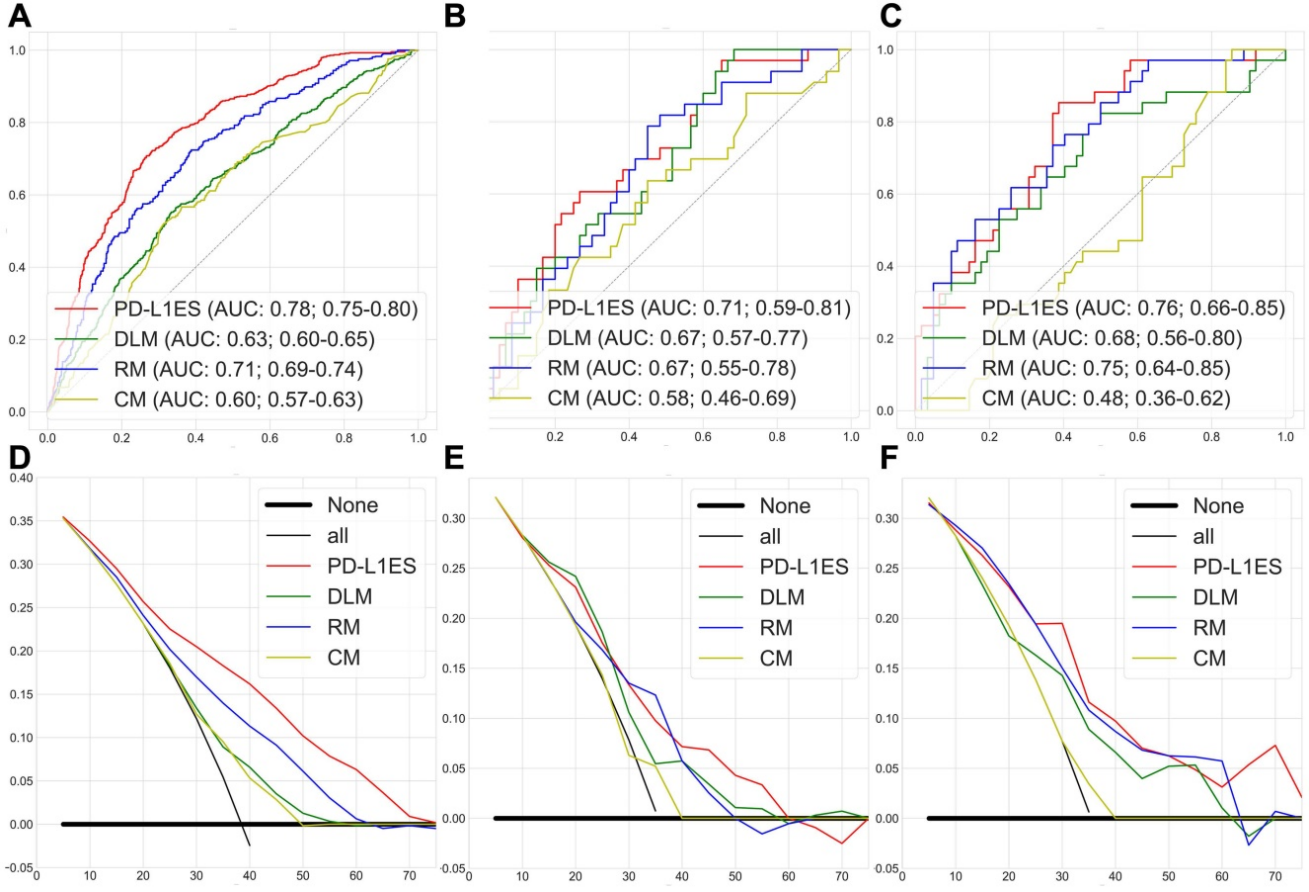
PD-L1 expression signature assessment results for high and low expression classification. (**A, B and C**) were the ROC curves of the training, validation, and test cohorts, respectively. (**D, E and F**) were the decision curves of the training, validation, and test cohorts, respectively. Among them, each figure contained the results of PD-L1 expression signature (PD-L1ES), deep learning model (DLM), radiomic model (RM) and clinical model (CM). They all showed that PD-L1 expression signature was superior to other models.

**Figure 2A-C** shows the ROC curves of PD-L1ES, where the AUCs of the training, validation and test datasets were 0.78 (95% CI: 0.75~0.80), 0.71 (95% CI: 0.59~0.81), and 0.76 (95% CI: 0.66~0.85), respectively. All results were significantly better than the DL, radiomic and clinical models (*P*<0.0001; ROC test was performed on the PD-L1 expression dataset). We concluded that PD-L1ES demonstrated good ability to distinguish between high and low expression of PD-L1 (≥ 50% and < 50%). In addition, the decision curves of the training and test cohorts further validated our conclusion (**Figure 2D-F**). We also found that PD-L1ES performed well in different layer thicknesses (greater than or equal to 5 mm and less than 5 mm) and X-ray tube current (the median is divided into two queues; cut-off point: 290) (**Supplementary Figure 2**). In addition, to evaluate the robustness of PD-L1ES, we randomly divided the validation and the test cohorts and re-established the signature. In the experimental verification results of the first random division, the AUC of the training, validation and test cohorts were 0.79 (95% CI: 0.77~0.82), 0.73 (95% CI: 0.61~0.85) and 0.76 (95% CI: 0.67~0.87). In the experimental verification results of the second random division, the AUC of the training, validation and test cohorts were 0.77 (95% CI: 0.74~0.80), 0.74 (95% CI: 0.60~0.86) and 0.75 (95% CI: 0.66~0.84). The results of two additional experiments revealed that PD-L1ES was more robust.

### Validation of PD-L1 expression signature in predicting the efficacy of immunotherapy

To explore the value of predicting the efficacy of immunotherapy using PD-L1 expression scores, we performed a Kaplan-Meier curve analysis based on PD-L1ES. The PD-L1ES was binarized with a cut-off of 0.66 obtained with X-tile. The group with PD-L1ES greater than 0.66 were termed the high PD-L1ES (high-risk) group, while the remaining were termed the low PD-L1ES (low-risk) group.

**Figure 3:**
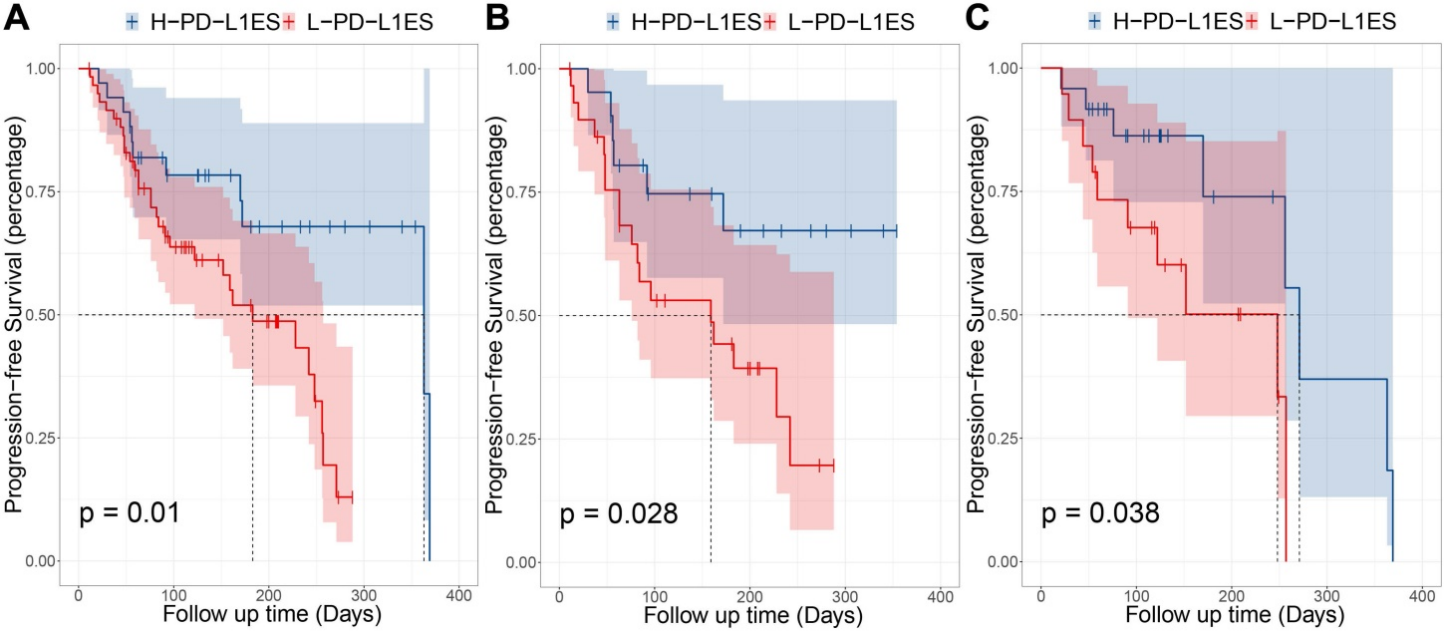
Prognostic performance in different subgroups, comparing high PD-L1 expression signature and low PD-L1 expression signature. (**A**) Progression-free survival (PFS) of patients relative to PD-L1 expression signature (high or low, as defined by the median value) in whole immunotherapy dataset. (**B**) PFS of patients in pembrolizumab combined with chemotherapy group. (C) PFS of patients in pembrolizumab-monotherapy group.

The univariable Cox analysis identified the binarized PD-L1ES as a significant prognostic variable (C-index: 0.66, 95% CI: 0.48~0.83; Hazard Ratio (HR): 2.57, 95% CI: 1.22~5.44; P = 0.010). In this study, low PD-L1ES was associated with improved PFS (**Figure 3**). The median PFS in the high-risk group was 183 days (95% CI: 122~257) and 363 days (95% CI: 363~) in the low-risk group.

Moreover, stratified analysis was performed based on the treatment regime. For patients who received immunotherapy, the low-risk and high-risk groups showed significant differences in PFS (*P* = 0.028). In patients administered immunotherapy combined with chemotherapy, a median PFS of 271 days was observed in the low-risk group compared to 248 days in the high-risk group (*P* = 0.038).

### Exploring the relationship between PD-L1 expression signature and clinical characteristics

We performed a stratified analysis for different clinical characteristics. Among them, we found that except for age (P_Age_ = 0.028), other clinical characteristics were not effective in stratifying patients into high and low-risk (P_Family history_ = 0.17; P_Smoking status_ = 0.22; P_Gender_ = 0.95) groups. The best cut-off value for age was 63. All Kaplan-Meier curves are shown in **Supplementary Figure 3.**

To further exploit the efficacy of PD-L1ES, multivariable Cox regression analysis was performed with a combination of signature and clinical characteristics. Clinical characteristics were not significant when the signature and each characteristic were combined separately (P_Family history_ = 0.064; P_Smoking status_ = 0.245; P_Gender_ = 0.374, P_Age_ = 0.088).

**Figure 4:**
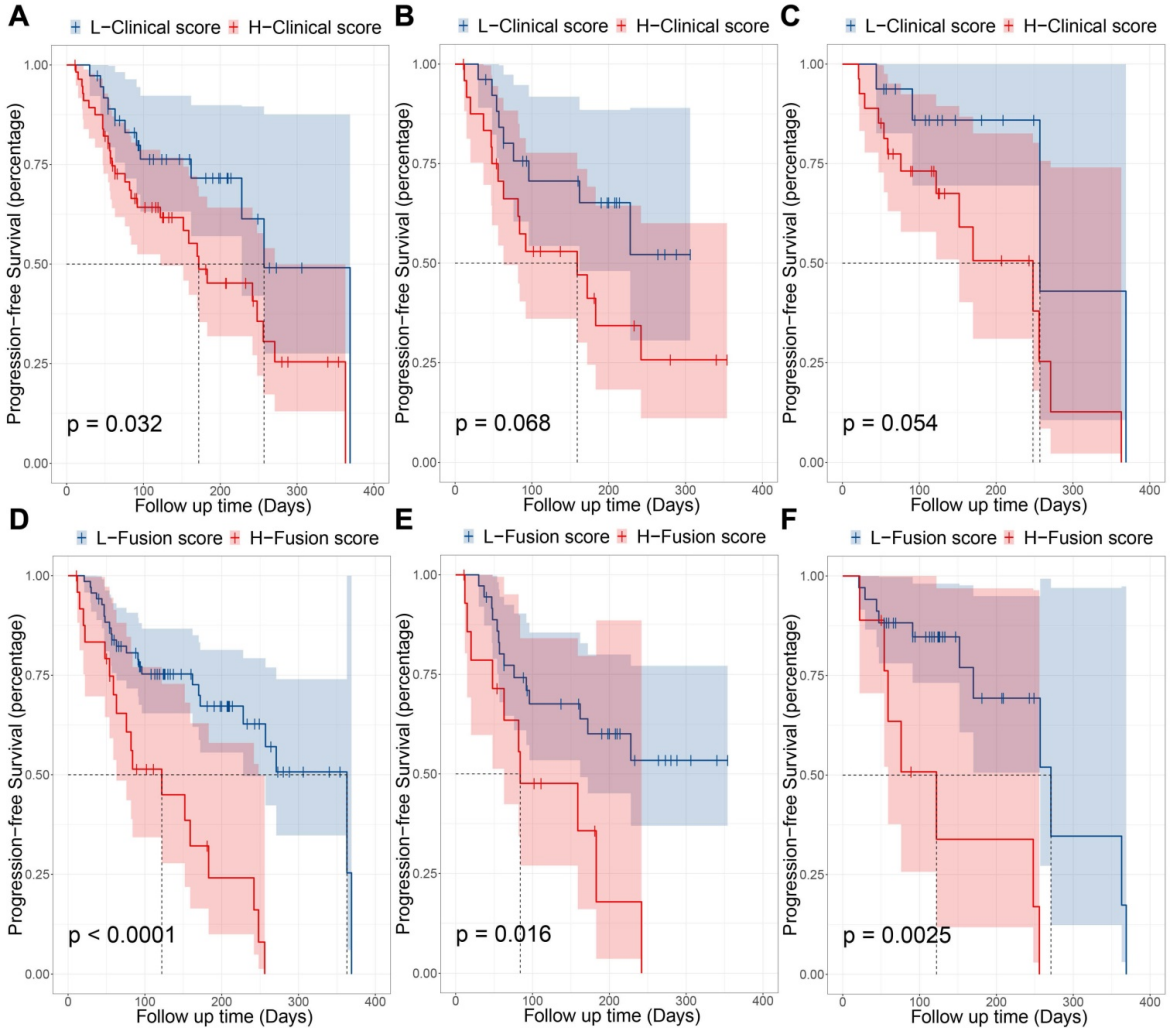
Prognostic performance of clinical model and fusion model. (**A, B and C**) were the KM curves which indicated progression-free survival (PFS) of patients relative to clinical model in entire immunotherapy cohort, pembrolizumab-monotherapy group and pembrolizumab combined with chemotherapy group, respectively. (**D, E and F**) were the KM curves which indicated PFS of patients relative to combination of PD-L1 expression signature (PD-L1ES) and clinical model in entire immunotherapy cohort, pembrolizumab-monotherapy group and pembrolizumab combined with chemotherapy group, respectively.

To explore the best predictive level of signature, we used all clinical characteristics to build a clinical model. Cox regression analysis was performed using a combination of signature and clinical models. We used Cox regression modeling with age, gender, smoking status, and family history. After using X-tile to select the best cut-off point (0.05 in this study), the clinical model classified patients into high-risk and low-risk groups (HR: 2.09, 95% CI: 1.05~4.18; P = 0.032) significantly, and the median PFS for high-risk and low-risk is 172 days and 257 days, respectively. Its prognostic stratification effect is not as good as PD-L1ES. Using the clinical model in combination with PD-L1ES for combinatorial analysis, we found that both variables were very significant (P_PD-L1ES_ = 0.0396; P_Clinical model_ = 0.010; Cut-off point = 0.05) in the combination of clinical model and PD-L1ES. Fortunately, the model also demonstrated strong stratification capabilities (HR: 3.53, 95% CI: 1.86~6.72; *P* < 0.001; Cut-off point = 1.78) and the median PFS has been significantly improved. The median PFS for high and low-risk groups was 122 days and 363 days, respectively. Results of the fusion model were superior to both the clinical and PD-L1ES models (**Figure 4**). Next, we performed the same subgroup analysis of the clinical model and fusion model as PD-L1ES. Results indicated that the clinical model was unable to significantly stratify patients in the immunotherapy group and immune combined chemotherapy group (P_Immunotherapy_ = 0.068; P_Immunotherapy combined chemotherapy_ = 0.054), while the fusion model demonstrated better performance than PD-L1ES in both groups (P_Immunotherapy_ = 0.016; P_Immunotherapy combined chemotherapy_ = 0.003).

### Visualization

**Figure 5:**
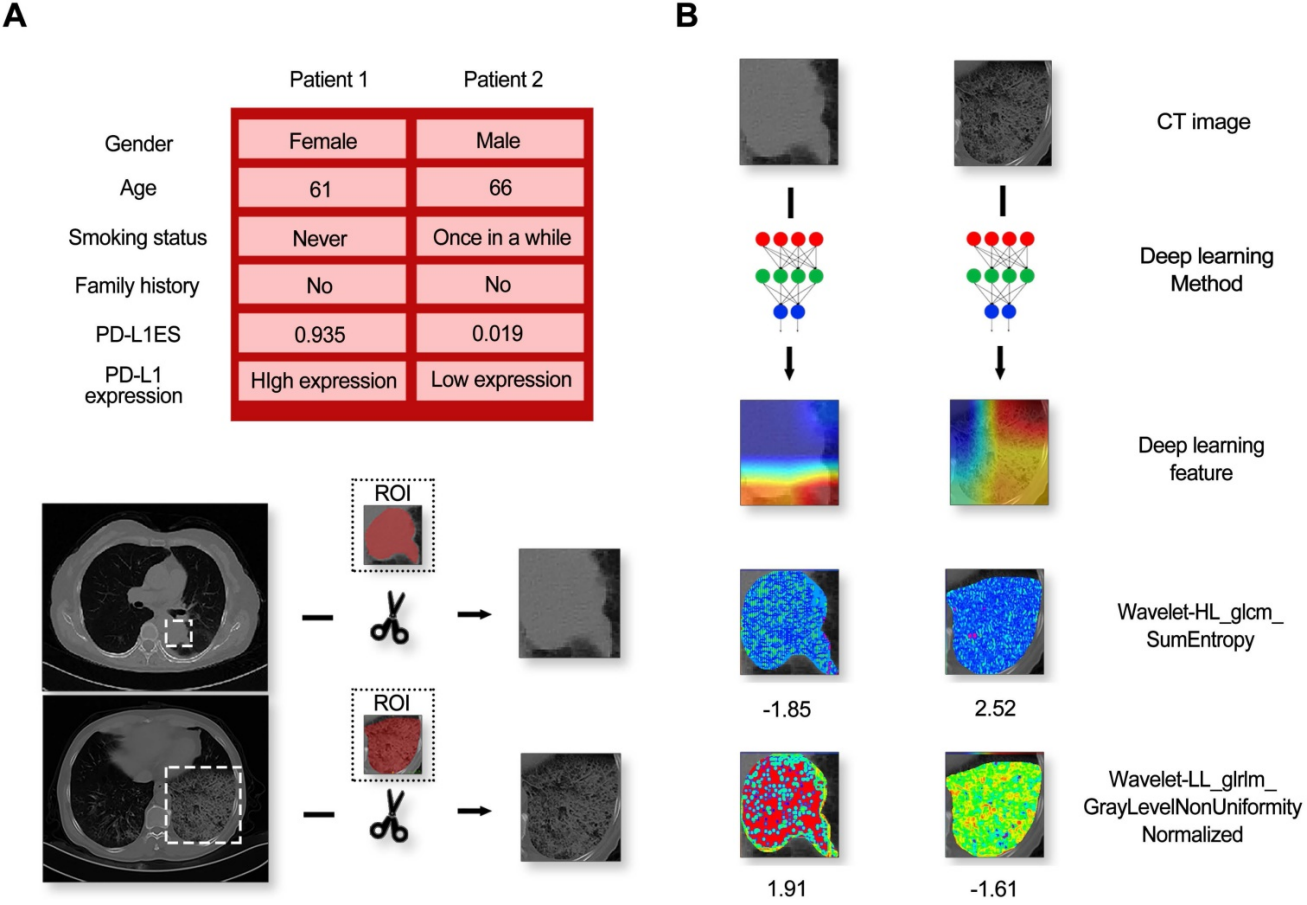
The information of patients and model feature visualization. (**A**) Contained a random sample of a high-expression patient and a low-expression patient and their corresponding CT images. (**B**) Indicated the visual images of the imaging features (including deep learning features and typical radiomic features).

A high-expression patient (Patient 1) and a low-expression patient (Patient 2) in the immunotherapy dataset are shown as an example. The class activation map was generated by the gradient of the deep learning to observe important regions that the model considers. At the same time, two representative radiomic features based on univariate analysis were also visualized in the ROI. Details of the patients and the corresponding visualization results are shown in **Figure 5.**

From this figure, we found that regardless of high and low expression, the primary observations of the deep learning model were located at the border of the lesion. In addition, huge texture differences in the lesion areas between two patients were seen from the visualization of the radiomic features, termed Wavelet-HL_glcm_SumEntropy and Wavelet-LL_glrlm_GrayLevelNonUniformityNormalized.

## Discussion

Radiomics is an emerging method that can convert medical images into quantitative data to profile tumor phenotypes 30,31. In our study, we established PD-L1ES via deep learning techniques to identify patients with high PD-L1 expression with high accuracy (AUCs ≥ 0.71), which could help to predict the efficacy of immunotherapy in patients with NSCLC.

Currently, IHC is the primary technique utilized to detect PD-L1 expression levels and has many limitations, including sampling bias due to intratumoral heterogeneity and dynamic characteristics of PD-L1 expression levels. As such, some noninvasive PET imaging novel radiotracers, including 89Zr-nivolumab, and 18F-BMS-98619210, demonstrated the predictive value of PD-L1 expression. However, they have not been clinically leveraged 32-34. Utilizing the commonly acquired PET/CT images, Jiang et al. assessed PD-L1 expression by radiomic features in NSCLC patients 35, achieving an AUC of 0.80 with only CT radiomic features in predicting PD-L1 (SP142) expression levels over 1%. Though this result is similar to that of our study (AUC_Training cohort_ = 0.78; AUC_Validation cohort_ = 0.71; AUC_Test cohort_ = 0.76), further prognostic validation of the predicted PD-L1 expression was not investigated. There are several benefits in assessing PD-L1 expression levels by radiomic-based signatures. First, its noninvasive manner outperformed IHC which demands tissue samples obtained from surgery or biopsy. Additionally, radiomic-based signatures can dynamically evaluate PD-L1 expression stratification and immune therapeutic efficacy, ultimately informing drug adjustment.

Several studies have evaluated associations between radiomics and other immunotherapy-related genomics or biology. Sun and colleagues evaluated the association between a radiomics-based biomarker of tumor-infiltrating CD8 cells and clinical outcomes of anti-PD-1 and PD-L1 treatment 36. They found that the gene expression signature of CD8 cells discriminated inflamed tumors from immune-desert tumors (AUC: 0.76). Furthermore, high baseline radiomic scoring was also associated with improved overall survival in patients treated with immunotherapy. Trebeschi and colleagues performed artificial intelligence (AI)-based characterization of primary and metastatic lesions from 203 patients using pretreatment CT imaging data to develop and validate a machine learning biomarker for response to immunotherapy 37. They found that when combining lesion-wide predictions, immunotherapy response was predicted with an AUC of 0.76 for NSCLC and melanoma (*P* < 0.001). However, various tumor types were included in these studies.

In our study, we first reported on PD-L1 expression prediction using a CT-based deep learning model in NSCLC patients. In addition, we generated visualization of the category activation map and radiomic features. We found that deep learning was more concerned with the marginal zone of the lesion area, and huge textural differences were observed from the visualization maps of texture features, named Wavelet-HL_glcm_SumEntropy and Wavelet-LL_glrlm_GrayLevelNonUniformityNormalized. This result confirmed that classification of high and low PD-L1 expression with deep learning features and quantitative radiomic features were complementary.

Our study has some limitations. First, it was a single-center study, and the predictive value of the obtained imaging biomarker has not been validated in other centers. Second, it was retrospective; therefore, potential bias may exist. In this study, we found that the higher the signature value, the greater the risk, which is contrary to common sense. The reasons for this situation can be summarized into two aspects. First, only a small number of patients were included in the immunotherapy dataset. Secondly, censored data existed in some patients. In addition, since only a minority of patients died in the follow-up period, we did not obtain median OS data. Thus, we are uncertain about the predictive value of this model on long-term efficacy of immunotherapy. In subsequent studies, we will include more immunotherapy patients, and use a combination of retrospective and prospective methods for signature verification and optimization.

## Conclusions

The deep learning model provides a noninvasive method for predicting high PD-L1 expression in tumors and for inferring clinical outcomes of immunotherapy. In addition, this deep learning model combined with clinical models improved prediction of the response to immunotherapy.

## Supplementary Material

Supplementary figures and tables.

## Figures and Tables

**Table 1:** Clinical characteristics of the PD-L1 expression dataset

Characteristics	Total (n = 939)	Training cohort (n = 750)	Validation cohort (n = 93)	*P* Value*	Test cohort (n = 96)	*P* Value**
Age, year, mean ± SD	58.8 ± 10.7	58.9 ± 10.7	58.0 ± 10.3	0.462	59.0 ± 11.3	0.892
**Gender, n (%)**				0.838		0.408
Male	576 (61.3)	455 (60.7)	58 (62.4)		63 (65.6)	
Female	363 (38.7)	295 (39.3)	35 (37.6)		33 (34.4)	
**Smoking status, n (%)**				0.938		0.409
Never	432 (46.0)	348 (46.4)	43 (46.2)		41 (42.7)	
Occasionally	374 (39.8)	300 (40.0)	36 (38.7)		38 (39.6)	
Constantly	95 (10.1)	74 (9.7)	11 (11.8)		10 (10.4)	
Unknown	38 (4.0)	28 (3.7)	3 (3.2)		7 (7.2)	
**Family history of cancer, n (%)**				0.686		0.437
No	764 (81.4)	610 (81.3)	79 (84.9)		75 (78.1)	
Yes	139 (14.8)	113 (15.1)	11 (11.8)		15 (15.6)	
Unknown	36 (3.8)	27 (3.6)	3 (3.2)		6 (6.3)	
**PD-L1 expression, n (%)**				0.988		0.995
≥ 50%	328 (34.9)	261 (34.8)	33 (35.5)		34 (35.4)	
< 50%	611 (65.1)	489 (65.2)	60 (64.5)		62 (64.6)	

Categorical data are shown as numbers (%) and continuous data as mean ± SD; *The* P* value is the test result of the training cohort and the validation cohort; **The *P* value is the test result of the training cohort and the test cohort.

## Abbreviations

AI: artificial intelligence
AUC: areas under the receiver operating characteristic curve
CI: confidence interval
CM: clinical model
CT: computed tomography
DL: deep learning
DLM: deep learning model
HR: hazard ratio
IBSI: The Imaging Biomarker Standardization Initiative
ICIs: immune checkpoints inhibitors
IHC: immunohistochemical
KNN: k-nearest neighbor
NSCLC: non-small cell lung cancer
OS: overall survival
PD-L1ES: PD-L1 expression signature
PFS: progression-free survival
RM: radiomic model
ROC: the receiver operating characteristic curve
ROI: region of interest
TPS: tumor proportion score
