# Supplementary material for: Assessing PD-L1 expression in non-small cell lung cancer and predicting responses to immune checkpoint inhibitors using deep learning on computed tomography images
Source: Theranostics. 2021 Jan 1;11(5):2098–107. doi: 10.7150/thno.48027 (PMC7797686; doi:10.7150/thno.48027)
Supplement: Supplementary file 1 — Supplementary figures and tables. [file thnov11p2098s1.pdf]

Supplementary tables and figures

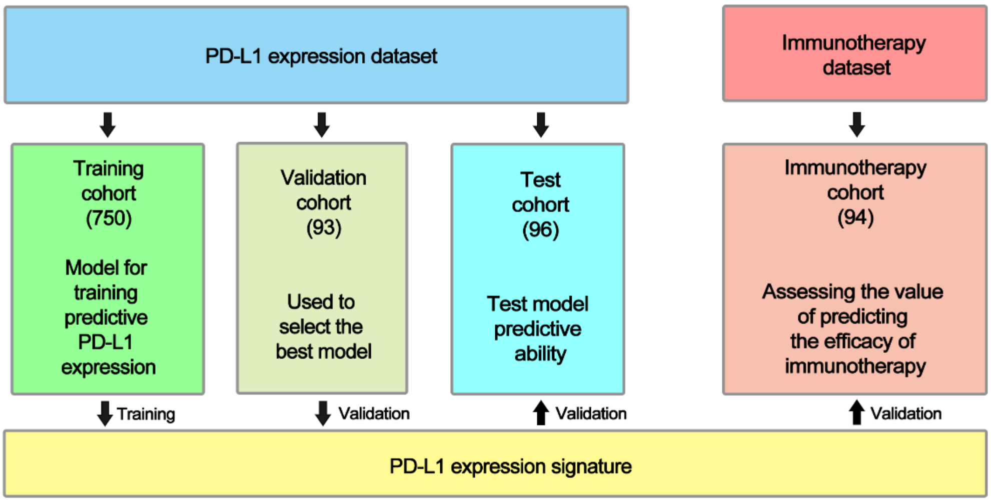


**Supplementary Figure 1.** Datasets and experimental flow chart in this study. A total of four datasets were included in this study, including the PD-L1 expression dataset (including training set, validation set and test set) related to obtaining PD-L1 expression signature and the immunotherapy dataset related to validated efficacy.

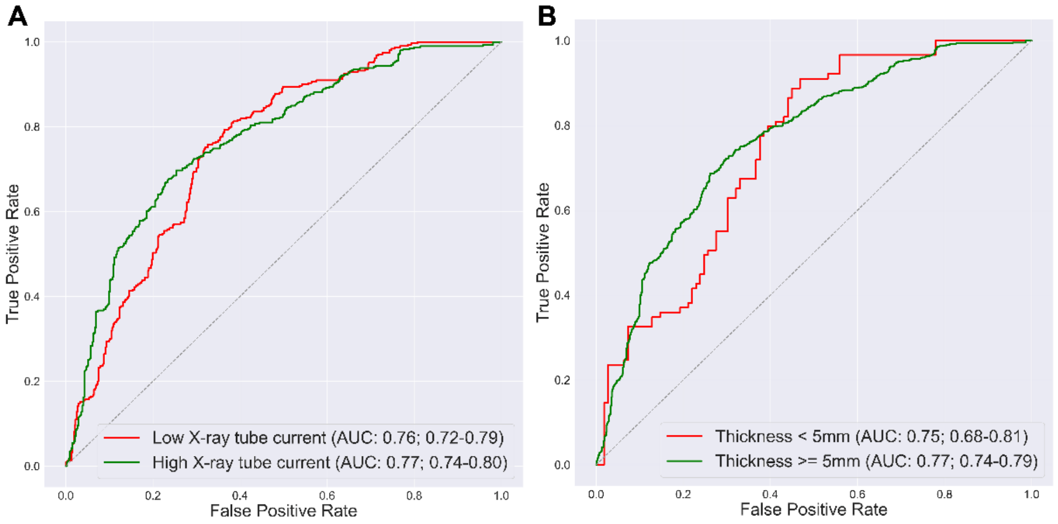


**Supplementary Figure 2.** Stratified analysis of high and low expression evaluation of PD-L1 expression signature based on CT scanning parameters. **(A)** and **(B)** were the ROC curves of stratified analysis which used PD-L1 expression signature based on X-ray tube current and slice thickness, respectively.

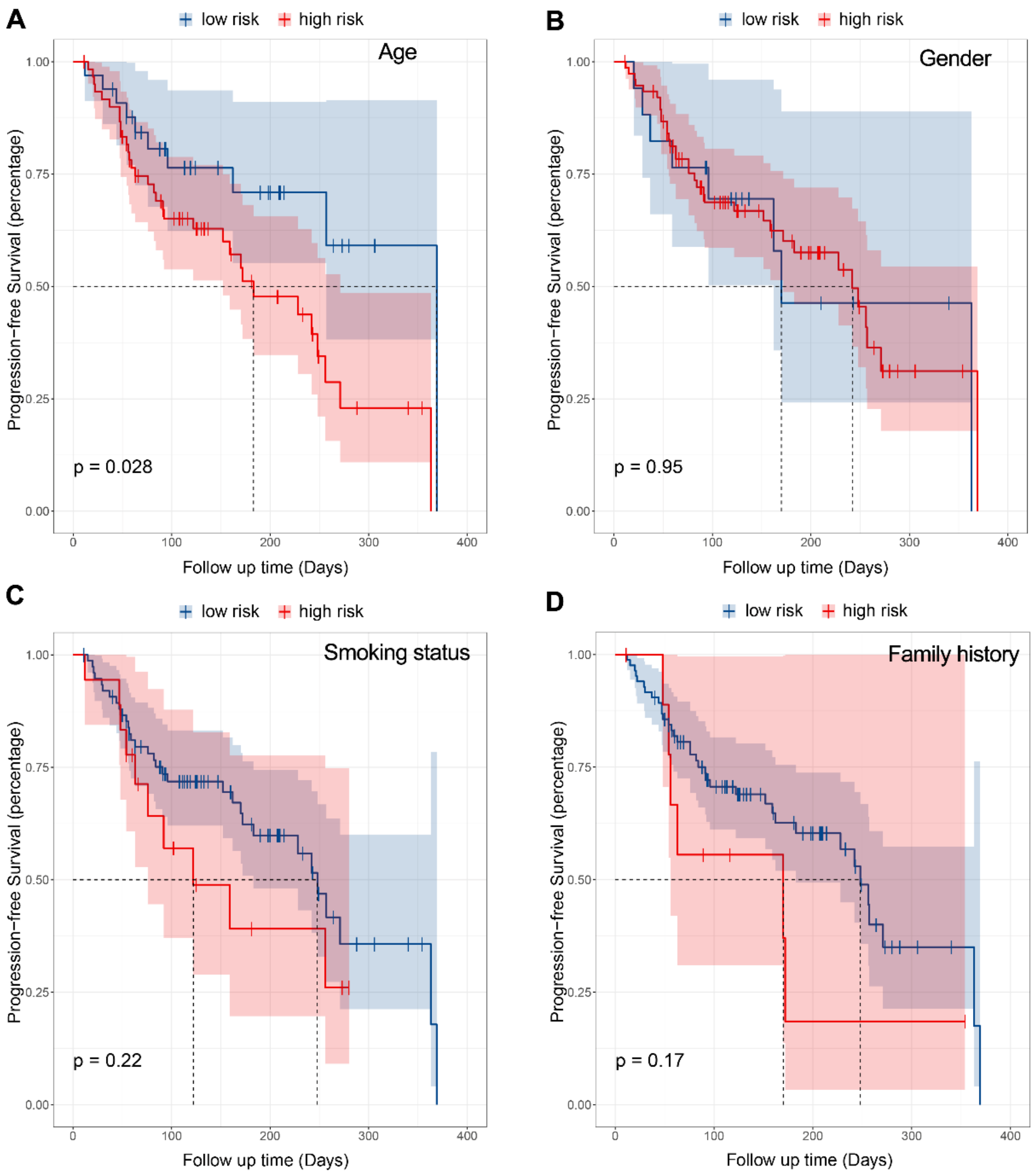


**Supplementary Figure 3.** Progression-free survival of patients according to different clinical characteristics models. **(A)**, **(B)**, **(C)** and **(D)** demonstrated the prognostic performance of different clinical characteristics models including age, gender, smoking status, and family history of tumor, respectively.

## Supplementary Table 1. Clinical characteristics of the immunotherapy dataset

| Characteristics | Total (n = 94) | High risk group (n = 60) | Low risk group (n = 34) | P Value |
|---|---|---|---|---|
| **Age, year, mean ± SD** | 60.7 ± 11.1 | 59.9 ± 9.3 | 61.1 ± 12.0 | 0.614 |
| **Gender, n (%)** | | | | 0.845 |
| **Male** | 77 (81.9) | 28 (82.4) | 49 (81.7) | |
| **Female** | 17 (18.1) | 6 (17.6) | 11 (18.3) | |
| **Smoking status, n (%)** | | | | 0.952 |
| **Never** | 21 (22.3) | 13 (21.7) | 8 (23.5) | |
| **Occasionally** | 55 (58.5) | 35 (58.3) | 20 (58.8) | |
| **Constantly** | 18 (19.1) | 12 (20.0) | 6 (17.6) | |
| **Family history of cancer, n (%)** | | | | 0.935 |
| **Yes** | 10 (10.6) | 6 (10.0) | 4 (11.8) | |
| **No** | 84 (89.4) | 54 (90.0) | 30 (88.2) | |
| **Therapy method, n (%)** | | | | 0.376 |
| **Immunotherapy + Chemotherapy** | 43 (45.7) | 30 (50.0) | 13 (38.2) | |
| **Immunotherapy** | 51 (54.3) | 30 (50.0) | 21 (61.8) | |
| **PD-L1 expression value by IHC** | | | | < 0.001 |
| **Mean** | 0.68 ± 0.18 | 0.79 ± 0.08 | 0.48 ± 0.14 | |
| **Range** | 0.17, 1.00 | 0.66, 1 | 0.17, 0.69 | |

Categorical data are shown as numbers (%) and continuous data as mean ± SD;

**Supplementary table 2.** Statistics on radiomic features

| Filter | Category | n | Filter | Category | n |
|---|---|---|---|---|---|
| original | shape | 14 | lbp-2D | firstorder | 18 |
| original | firstorder | 18 | lbp-2D | texture | 75 |
| original | texture | 75 | lbp-3D-m1 | firstorder | 18 |
| wavelet-LH | firstorder | 18 | lbp-3D-m1 | texture | 75 |
| wavelet-LH | texture | 75 | lbp-3D-m2 | firstorder | 18 |
| wavelet-HL | firstorder | 18 | lbp-3D-m2 | texture | 75 |
| wavelet-HL | texture | 75 | lbp-3D-k | firstorder | 18 |
| wavelet-HH | firstorder | 18 | lbp-3D-k | texture | 75 |
| wavelet-HH | texture | 75 | logarithm | firstorder | 18 |
| wavelet-LL | firstorder | 18 | logarithm | texture | 75 |
| wavelet-LL | texture | 75 | square | firstorder | 18 |
| exponential | firstorder | 18 | square | texture | 75 |
| exponential | texture | 75 | squareroot | firstorder | 18 |
| gradient | firstorder | 18 | squareroot | texture | 75 |
| gradient | texture | 75 | | | |
